# Disulfide-mediated tetramerization of TRAP1 fosters its antioxidant and pro-neoplastic activities

**DOI:** 10.1016/j.redox.2025.103677

**Published:** 2025-05-13

**Authors:** Fiorella Faienza, Claudio Laquatra, Matteo Castelli, Gianmarco Matrullo, Salvatore Rizza, Federica Guarra, Azam Roshani Dashtmian, Alessia Magro, Paola Giglio, Chiara Pecorari, Lavinia Ferrone, Elisabetta Moroni, Francesca Pacello, Andrea Battistoni, Giorgio Colombo, Andrea Rasola, Giuseppe Filomeni

**Affiliations:** a Department of Biology, University of Rome Tor Vergata, Via della Ricerca Scientifica, I-00133, Rome, Italy; b Department of Biomedical Sciences, University of Padova, viale G. Colombo 3, I-35131, Padova, Italy; c Department of Chemistry, University of Pavia, via Taramelli 12, Pavia, I-27100, Italy; d Redox Biology, Danish Cancer Institute, Strandboulevarden 49, DK-2100, Copenhagen, Denmark; e SCITEC-CNR, via Mario Bianco 9, I-20131, Milano, Italy

**Keywords:** Oxidative stress, Mitochondria, Cysteine, Tumorigenesis, Metabolism

## Abstract

The mitochondrial chaperone TRAP1 exerts protective functions under diverse stress conditions. It induces metabolic rewiring and safeguards cancer cells from oxidative insults, thereby contributing to neoplastic progression.

TRAP1 works as a homodimer, but recent evidence indicated that it forms tetramers whose effects remain elusive. Here, we find that TRAP1 generates redox-sensitive tetramers via disulfide bonds involving cysteines 261 and 573. TRAP1 tetramerization is elicited by oxidative stress and abrogated upon expression of the double C261S/C573R mutant. In cancer cells, the TRAP1 C261S/C573R mutant is unable to inhibit the activity of its client succinate dehydrogenase and to confer protection against oxidative insults, thus hampering the invasiveness of aggressive sarcoma cells.

Overall, our findings indicate that TRAP1 undergoes tetramerization in response to oxidative stress and identify C261 and C573 as critical for TRAP1 structural rearrangement and functions.

## Graphical abstract

**Figure:**
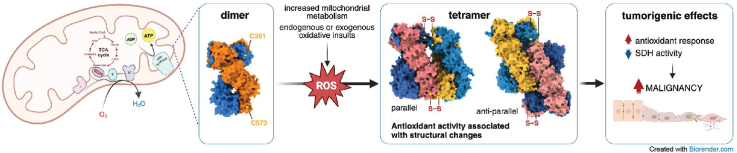

## 1 Introduction

TRAP1, the mitochondrial paralog of HSP90 molecular chaperones, is a crucial homeostatic regulator that protects cells from the noxious effects of metabolic changes and oxidative stress [1,2]. Under hypoxic conditions it stabilizes the transcription factor HIF1α, which in turn up-regulates TRAP1 expression in a feed-forward circuit [3]. The TRAP1/HIF1α axis tunes the bioenergetic activity of mitochondria to the availability of oxygen, mastering cell adaptations to the oxygen paucity that can occur during embryogenesis, ischemia or tumor accrual [1,[3], [4], [5], [6], [7]] and that can propel dyshomeostatic redox conditions [8]. Indeed, in several tumor types, TRAP1 expression correlates with aggressiveness and its ablation or pharmacological inhibition hamper neoplastic growth [9].

Multiple functions of TRAP1 can confer a survival and growth advantage to neoplastic cells exposed to oxidative damage. By down-regulating OXPHOS through interactions with the respiratory complexes cytochrome *c* oxidase [10] and succinate dehydrogenase (SDH) [11,12], TRAP1 can attenuate mitochondrial ROS generation. It also hinders the harmful effects of oxidative stress by inhibiting the mitochondrial permeability transition pore (mPTP) [13], a channel whose lethal opening can be elicited by a surge in oxidants [14]. However, changes in ROS levels have dichotomic effects on tumor progression, either exerting anti-neoplastic effects by cell death induction, or enhancing tumor malignancy by an increase in genomic instability [8]. This could explain why context-specific exceptions to the pro-neoplastic function of TRAP1 have been reported, where its expression is associated with a diminished neoplastic progression [2]. Hence, deciphering the molecular mechanisms of interaction between TRAP1 and its binding partners, called clients, and understanding how structural changes of TRAP1 influence its activity, are of paramount importance to clarify the biological outputs of TRAP1 engagement during tumor progression and other pathophysiological conditions characterized by changes in ROS levels.

The activity of TRAP1 in tumor models is regulated by post-translational modifications (PTMs). Phosphorylation on Tyr and Ser residues enhances TRAP1 inhibitory effects on OXPHOS complexes [10,15], whereas its interaction with the deacetylase SIRT3 favors stemness and malignancy in a glioma model [16]. Moreover, TRAP1 *S*-nitrosylation decreases its chaperone activity and primes it for degradation, enhancing cell sensitivity to death stimuli [[17], [18], [19]] and suggesting that via these redox-dependent modifications TRAP1 could sense fluctuations in ROS levels and act as a redox antenna.

Altogether, TRAP1 PTMs could prompt changes in the protein structure or dynamics that reverberate in variations of its activity, flexibly shaping it to fluctuations of intracellular conditions. On the same line, TRAP1 could aggregate multimeric protein complexes in tumor cell mitochondria, thus finely tuning whole signaling axes. Indeed, TRAP1 chaperone functions require formation of a homodimer that utilizes ATP to couple conformational changes with client remodeling [20], but cryo-EM observations indicate that TRAP1 can also form tetramers as dimers of dimers with different conformations [21] that could be posed to interact with large protein assemblies under specific conditions. Accordingly, formation of TRAP1 tetramers increases following perturbations in OXPHOS, a biochemical pathway that requires the concerted activity of large respiratory complexes [22]. However, it is unclear what triggers formation of TRAP1 tetramers, what are the effects of tetramerization on its chaperone activity, and if tetramers can regulate any specific biological function.

Here we demonstrate that TRAP1 can form tetramers following different oxidative stimuli via disulfide bridges involving C261 and C573, and that mutations of these residues affect TRAP1 activity and reduce its tumorigenic potential.

## 2 Materials and Methods

### 2.1 Plasmids

TRAP1 WT and redox mutants (C501S, C527A, C501S/C527A, C261S, C573R, C261S/C573R) were cloned in pET-26b(+) plasmid for bacterial expression and in pcDNA3.1(+)-C-HA plasmid for mammalian expression using the Gene Synthesis & DNA Synthesis service from GenScript Biotech. TRAP1 WT in pBABE vector was mutagenized to produce redox mutants (C261S, C573R, C261S/C573R) using the following primers: C261S 5′-CACCTGAAATCCGACTCCAAGGAGTTTTCCAGC-3′, C573R 5′-CAGGTCCCCAGCCGCCGAGCGCCTATCAGAGAA-3’. All pBABE plasmids were used for viruses' production. Plasmids coding for SOD2 (cloned in pcDNA3 vector) and M-CAT (cloned in pCAGGS vector) were obtained as reported in Ref. [17].

### 2.2 Production of recombinant proteins

Recombinant TRAP1 (WT and redox mutants) was produced in BL21(DE3) *E. coli* cells after 3 h-induction with 1 mM IPTG (Isopropil-β-d-tiogalactopyranoside, VWR). Protein purifications were performed using Ni-NTA resin (Quiagen) according to manufacturer's instructions. Proteins release from the resin packed in a FPLC column was achieved using a linear imidazole gradient generated by mixing *buffer A* (50 mM Phosphate Buffer, 250 mM NaCl, 10 mM imidazole, 10 mM β-mercaptoethanol, pH 7.8) and *buffer B* (buffer A containing 250 mM imidazole). Eluted fractions containing recombinant proteins were then collected and combined. After purification, the proteins were dialyzed in the *storage buffer* (50 mM Tris-HCl, 150 mM NaCl, 1 mM DTT, 1 mM EDTA, pH 7.5) and stored into aliquots.

### 2.3 Redox treatments

Purified TRAP1 (WT and redox mutants) were dialyzed in *storage buffer*. Before treatments, DTT was removed by Zeba Spin Desalting Columns (Thermo Fisher Scientific) and proteins transferred in a *reaction buffer* (150 mM NaCl, 50 mM TRIS, pH 7.5). Oxidation and reduction were performed at 37 °C by incubations with 10 nM-to-100 μM H_2_O_2_ (Sigma Aldrich) and 50 mM DTT (Sigma Aldrich), respectively.

### 2.4 Non-reducing SDS-PAGE

After treatments with oxidants or reductants, proteins were denatured with sample buffer (NuPAGE LSD sample buffer, Thermo Fisher Scientific), with or without the reducing agent (NuPAGE Sample reducing agent, Thermo Fisher Scientific), and loaded on gel for SDS-PAGE. Proteins were visualized by Coomassie Brilliant Blue staining (Sigma Aldrich) or with Stain-free technology (Bio-Rad Laboratories).

### 2.5 Gel filtration chromatography

The quaternary structure of WT and C261S/C573R mutant TRAP1 protein was analyzed by size-exclusion chromatography using the prepacked column Superdex 200 HR 10/30 (Amersham Pharmacia Biotech). Before loading, the buffer was changed by Zeba Spin Desalting Columns (Thermo Fisher Scientific) to remove DTT. One hundred and fifty mg of each protein sample was applied to the column and eluted with 20 mM TRIS, 150 mM NaCl, pH 7. Fractions of 0.5 ml were collected and analyzed by SDS-PAGE to verify that all the observed elution peaks were due to TRAP1. The apparent molecular weight of the different forms of TRAP1 was determined using a calibration line generated by eluting standard proteins of known molecular weight in the same buffer.

To study the effect of the oxidant treatment on the quaternary structure of WT and cysteine mutants of TRAP1, each protein was incubated with 100 μM H_2_O_2_ and subjected to gel filtration.

### 2.6 Structural models

Prior to docking calculations, the Cryo-EM structure of tetrameric TRAP1 (PDB: 7KLV(https://doi.org/10.2210/pdb7KLV/pdb)) was subjected to preparation steps. The structure was preprocessed with protein preparation wizard tool in Maestro. Missing loops were reconstructed accordingly to UniProt Q12931 with crosslink in Schrodinger Maestro suite 2022–4 (www.schrodinger.com). Missing loops in protomer A were residues 335–360, 558–573 and 631–637. Missing loops in protomer B were residues 357–361, 558–573 and 625–630. Subsequently, the resulting loops underwent a minimization step with Prime until the RMS gradient convergence of 0.01 kcal/mol/Å was reached (solvation model: VSGB; Force field: OPLS4).

Protein-protein docking was carried out using PIPER in Schrodinger Maestro suite 2022–4 (www.schrodinger.com). Distance constraints were used as follows.

• Parallel configuration was obtained with C261(protomer-A)-C261(protomer-D) and C573(protomer-B)-C573(protomer-C) constraints.
• Antiparallel configuration was obtained with C261(protomer-A)-C573(protomer-C) and C261(protomer-D)-C573(protomer-B) constraints.

For each configuration, the best pose was optimized through minimization in explicit solvent (TIP3P water) with Prime (100ps). The last frame of minimization was used as starting conformation for 10 ns of MD (without any constraints) using the Desmond engine with default parameters [23], and TIP3P water as a solvent model [24]. The last frame of the MD simulations is shown in Fig. 2.

### 2.7 Cell lines

A375 and HeLa cell lines were obtained from the American Type Culture Collection (ATCC, Virgnia, USA). Malignant peripheral nerve sheath tumor cells (MPNST) were established from neurofibromin 1 (Nf1)-deficient skin precursors (SKP) and were kindly provided by Dr. Lu Q. Le, University of Texas Southwestern Medical Center, Dallas, TX, and have been characterized in Sanchez et al. [25]. MEF Nf1^−/−^ were characterized in Masgras et al. [15]. Cells were grown Dulbecco's Modified Eagle's Medium (DMEM) and RPMI-1640 supplemented with 10 % fetal bovine serum (FBS, Thermo Fisher Scientific) 100 U/ml and 1 % penicillin/streptomycin (Euroclone). All cells were cultured at 37 °C in a humidified atmosphere containing 5 % CO_2_.

### 2.8 Generation of MPNST and MEF mutant cells

pBABE vectors containing both the WT and the C261S/C573R form of TRAP1 were used to stably transfect MPNST/MEF *Nf1*^−/−^ TRAP1 KO cells previously generated by Sanchez-Martin et al. [25]. pBABE vectors were co-transfected with packaging plasmids PMD2.G and psPAX2 into HEK 293 cells for viral production. Recombinant virus was used to infect MPNST, which were subsequently selected with 0.75 mg/ml G418 (Sigma Aldrich). Expression of recombinant protein was evaluated with Western blot analysis.

### 2.9 Generation of TRAP1-depleted HeLa cells

Stable TRAP1-depleted HeLa cells were generated by transfection of a TRAP1 3′UTR-targeting pre-miRNA-containing plasmid as previously described [26]. The plasmid was generated by cloning the following constructs in pcDNA6.2-GW/EmGFP-miR vector using the BLOCK-iT™ Pol II miR RNAi Expression Vector Kit (Life Technologies): Top Strand: 5′-TGCTGAGGTAAATAAAGCTCAAGGAGGTTTTGGCCACTGACTGACCTCCTTGATTTATTTACCT-3'; Bottom Strand: 5′-CCTGAGGTAAATAAATCAAGGAGGTCAGTCAGTGGCCAAAACCTCCTTGAGCTTTATTTACCTC-3'. Upon transfection, cells were cultured for 4 weeks in the presence of Blasticidin S and GFP-expressing cells were enriched twice using BD FACSMelody Cell Sorter (Beckman Coulter).

### 2.10 Cell treatments

For redox treatments cells were incubated with 100 μM H_2_O_2_ or with 50 mM DTT for 10 min in PBS at room temperature. Other treatments were performed as follow: a) diamide (Sigma Aldrich D3648) was used 1 mM for 30 min; b) oligomycin A (Calbiochem 495455) was used 10 μM for 4 h; c) cisplatin (CDDP, Sigma Aldrich) was used 10 μM for 24 h; d) buthionine sulfoximine (BSO, Sigma Aldrich) was used 100 μM for 24 h; e) dimethylsuccinate (DMS, SAFC W23,960-7-K) was used 2 mM for 10 days; f) MitoPQ (Cayaman chemical 36541) was used 1 μM for 2 h); g) H_2_O_2_ (Sigma Aldrich) was used 400 μM overnight; h) *N*-acetyl-l-cysteine (NAC, Sigma Aldrich A9165) was used 10 mM for 24 h or 5 mM every 2 days for 10 days in focus forming assay.

### 2.11 Generation of hypoxia-reoxygenation conditions

sMPNST cells were subjected to hypoxic conditions (0.5 % O_2_ Baker in vivO_2_ hypoxic workstation) for 2 h, followed by reoxygenation at atmospheric oxygen levels for 10 min. Subsequently, mitochondria were isolated as described in the following section and analyzed by Western blotting.

### 2.12 Mitochondrial isolation for tetramer detection

Mitochondria were isolated upon cell disruption with a glass-Teflon potter in RLM buffer composed of 250 mM sucrose, 10 mM Tris-HCl, 0.1 mM EGTA‐Tris, pH 7.4. Sixty μg of mitochondria were pulled down at 8200 rpm for 10 min at 4 °C and re-suspended in RLM buffer. To induce tetramer formation, mitochondria were treated with 100 μM H_2_O_2_ or 1 mM diamide, while 150 mM of DTT was used as negative control. All treatments were performed for 10 min at room temperature. After treatments, mitochondria were pulled down at 8200 rpm for 10 min, lysed in EB buffer (composed of NaCl 150 mM, Tris 20 mM pH 7.4, EDTA 5 mM, 1 % Triton X-100, Glycerol 10 %) and incubated on ice for 20 min and clarified at 18, 000×*g* for 20 min. Supernatant was collected and used for Western blot analyses.

### 2.13 Nuclei isolation for NRF2 nuclear translocation analysis

Cells were suspended in fractionation buffer (10 mM Tris-HCl pH 8.0, 250 mM sucrose, 1 mM EDTA, 4 mM MgCl_2_, 1 % Triton X-100, 0.5 mM DTT, plus protease inhibitors). After 30 min in ice, samples were centrifuged at 10,000×*g* for 10 min at 4 °C to isolate nuclei. Supernatants were kept as cytosol while pellet, containing nuclei, were washed three times with fractionation buffer and 10,000×*g* centrifuges for 10 min at 4 °C. Then, nuclei were lysed with whole lysis buffer (10 % glycerol, 2 % SDS, 10 mM Tris-HCl pH 6.8) and subjected to Western blot analysis.

### 2.14 Western blot analysis

Cells were lysed with Cell Lytic (Sigma Aldrich) plus protease inhibitors (Protease Inhibitors Cocktail, Sigma-Aldrich) and phosphatases inhibitors (1 mM Sodium fluoride and 1 mM Sodium orthovanadate, Sigma-Aldrich). After clarification protein extracts were quantified with DC protein assay (Bio-Rad Laboratories) protocol and then processed as described above (*Non reducing SDS-PAGE*). After SDS-PAGE, protein complexes were transferred onto nitrocellulose or PVDF membranes (GE HealthCare Amersham) and probed with anti-TRAP1 (anti-murine TRAP1 BD and anti-human TRAP1 SC-73604 and SC-13557), anti-citrate synthase (Abcam ab96600), anti NRF2 (Cell Signaling Technology, 12721), anti-histone H3 (Cell Signaling Technology, 4499), anti-tubulin (Santa-Cruz Biotechnology, SC-5286) antibodies.

### 2.15 Measurement of succinate:coenzyme Q reductase (SQR) activity of SDH

Succinate dehydrogenase (SDH) activity was measured as already described by Sanchez-Martin et al. [25]. Briefly, samples were collected and lysed at 4 °C in a buffer composed of 25 mM potassium phosphate, pH 7.2, and 5 mM MgCl_2_ containing protease and phosphatase inhibitors. Total lysate was quantified with BCA protein assay Kit (Thermo-Scientific), and 40 μg of protein per trace was incubated 10 min at 30 °C in the presence of 20 mM sodium succinate and 10 mM alamethicin. After incubation, a mix composed of 5 mM sodium azide, 5 μM antimycin A, 2 μM rotenone, and 65 μM Coenzyme Q1 was added. SDH activity was measured by following the reduction of 2,6-dichlorophenolindophenol (DCPIP) at 600 nm (ε = 19.1 nM^−1^ cm^−1^) at 30 °C. Each measurement was normalized for protein amount.

### 2.16 Measurement of ROS levels

ROS levels were evaluated on MPNST TRAP1-KO cells expressing the *wild-type* and C261S–C573R variants of human TRAP1 treated or not with mitoPQ 1 μM for 2 h. Upon treatment, cells were stained for 30 min with 5 μM MitoSOX (Invitrogen M36008) and analyzed with a Fortessa X-20 flow cytometer (Becton Dickinson).

### 2.17 Measurement of protein carbonylation

Cells were lysed with Cell Lytic (Sigma Aldrich) plus 50 mM DTT and protease inhibitors cocktail as described above in the section of Western blot analysis. 20 μg of proteins was used to derivatize protein carbonyls with DNPH (2,4-dinitrophenylhydrazine) by using OxyBlot protein oxidation detection kit (Millipore) accordingly to manufacture's protocol.

### 2.18 Real-time PCR

RNA was extracted by ReliaPrep RNA Cell Miniprep System (Promega) and cDNA was generated using the GoScript Reverse Transcription System (Promega) following manufacturer's protocols. Real-time PCR was performed using the iTAQ universal SYBR Green Supermix (Bio-Rad Laboratories) on a VIAA 7 real-time PCR System (Applied Biosystems). Changes in mRNA levels were expressed relative to a control after normalizing to the internal standard 60S ribosomal protein L34 (RPL34), calculated with the 2^−DDct^ method (PMID: 11846609).

#### 2.18.1 Primers sequence

**mRPL34**: FW_GGTGCTCAGAGGCACTCAGGATG, RV_GTGCTTTCCCAACCTTCTTGGTGT

**mGCLm**: FW_CGCACAGCGAGGAGCTTCGG, RV_CTCCACTGCATGGGACATGGTGC

**mNQO1**: FW_CGCCTGAGCCCAGATATTGT, RV_GCACTCTCTCAAACCAGCCT

### 2.19 *In vitro* tumorigenicity assay

Focus forming assays were performed on MPNST TRAP1-KO cells expressing the wild-type and C261S–C573R variants of human TRAP1. Cells were grown in 12-well culture plates in DMEM medium supplemented with 10 % fetal bovine serum. When cells reached sub-confluence, serum concentration was decreased to 1 %. After 10 days, plates were washed in PBS, fixed in methanol for 30 min, stained with GIEMSA solution for 1 h and analyzed with ImageJ software.

### 2.20 Spheroid formation and *in vitro* migration assays

For spheroid generation, 5000 MPNST cells were plated in round-bottom 96 well plates, centrifuged at 100×*g* for 1 min at room temperature and incubated at 37 °C 5 % CO_2_. After 48 h, 50 μl of DMEM was replaced with same volume of Matrigel (Corning 356231), centrifuged at 100×*g* for 1 min at 4 °C, and incubated at 37 °C, 5 % CO_2_. After 5 days, cell spreading was analyzed with ImageJ software by calculating the area of spheroids branches.

### 2.21 Cell proliferation assay

To assess the proliferation of sMPNST cells expressing either the WT or the C261S/C573R human form of TRAP1, 10000 cells were plated in a 12-well plate in standard DMEM. After 24 h from plating, quantification was performed up to 96 h, as indicated in Tiberti et al., 2022 [27]. Cells were stained for 15 min using a solution of 0.025 % crystal violet and 20 % methanol. Cells were then washed with water and decolorized with 100 % methanol for 30 min. The data are represented as absorbance measured at 595 nm, which is directly proportional to the number of cells present in each well.

## 3 Results

### 3.1 A disulfide bond linking C261 and C573 drives TRAP1 tetramerization

**Fig. 1:**
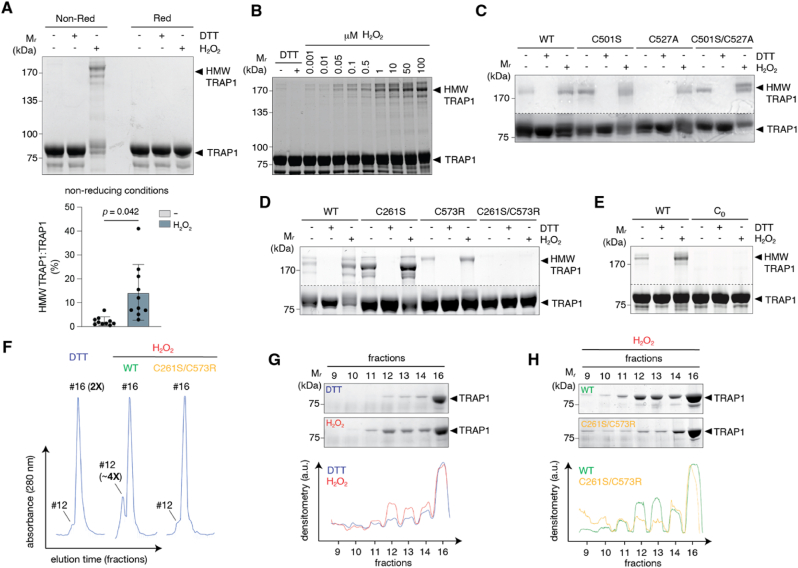
TRAP1 forms redox-sensitive tetramers. **A.***Wild type* (WT) recombinant human TRAP1 exposed to 100 μM H_2_O_2_ and/or 10 mM DTT for 30 min and analyzed by non-reducing or reducing SDS-PAGE (*top*). Densitometric analyses of n = 10 independent experiments (*p* = 0.042). Data are expressed as mean ± S.D. Paired *t*-test was applied for statistical analyses (*bottom*). **B.** WT TRAP1 incubated with increasing concentrations of H_2_O_2_ (from 1 nM to 100 μM). **C-E**. WT and different Cys-mutant treated as in A. Gels were reduced to show only the high-molecular-weight (HMW) form of TRAP1 (above 170 kDa) and the monomeric TRAP1 (∼80 kDa). An horizontal dotted line stands for this digital cut. **F.** Gel-filtration chromatograms of recombinant TRAP1 (WT and C261S/C573R double mutant) exposed to 100 μM H_2_O_2_ or 10 mM DTT for 30 min. Fraction #12 refers to tetrameric TRAP1. Fraction #16 refers to dimeric TRAP1. **G, H.** Representative SDS-PAGE and band densitometry of fractions obtained by gel-filtration chromatography shown in E.

TRAP1 is known to respond to elevated ROS levels and to exhibit antioxidant functions, although the precise structural changes underlying these functions remain poorly characterized. To investigate the effects of oxidative conditions on TRAP1, we utilized a recombinant human TRAP1 protein lacking the mitochondrial import sequence, thus mimicking the mature form of the chaperone. Under non-reducing conditions, TRAP1 primarily migrated as a band with an apparent molecular mass of about 80 kDa. However, upon exposure to H_2_O_2_, an additional band emerged just above 170 kDa (Fig. 1A), suggesting that H_2_O_2_ treatment induced disulfide bridge formation linking two TRAP1 monomers (Fig. 1A). Interestingly, the band was already faintly detectable starting from 50 nM H_2_O_2_ but became progressively more prominent at concentrations in the range of μM, reaching a maximum at 100 μM (Fig. 1B). This concentration was therefore used for subsequent *in vitro* experiments. At this stage, however, it was unclear whether these disulfides were intra- or intermolecular.

Human TRAP1 contains four cysteine residues (C261, C501, C527, and C573). Previous studies have demonstrated that two of these cysteines, C501 and C527, are located in close proximity within an internal region and can form an intramolecular disulfide bond upon C501 *S*-nitrosylation [[17], [18], [19]]. Notably, no one of the four cysteine residues are positioned at the dimerization interface of TRAP1, which argues against the possibility that the H_2_O_2_-induced disulfide bonds link two monomers within the same TRAP1 dimer. To assess the role of these cysteines in forming high-molecular-weight TRAP1 structures, we generated mutants at each cysteine and evaluated their capacity to generate gel-retarded TRAP1 complexes upon H_2_O_2_ exposure. All the single mutants, along with the C501S/C527A double mutant, retained the ability to form high-molecular-weight complexes upon H_2_O_2_ treatment, and such complexes were disrupted by the thiol-reducing agent dithiothreitol (DTT) (Fig. 1C and D). In contrast, both the C261S/C573R mutant (Fig. 1D) and the cysteine-null (C_0_) variant (Fig. 1E) lost this ability. These results suggest that H_2_O_2_ exposure induces the formation of high-molecular-weight TRAP1 structures through disulfide bridges involving C261 and C573, likely linking two distinct TRAP1 dimers to form oligomeric structures, reasonably TRAP1 tetramers, in agreement with what was previously described [22].

To test this hypothesis, we performed gel filtration chromatography on native recombinant TRAP1. When disulfide bonds were reduced by DTT, TRAP1 eluted at fraction 16, with an observed-to-theoretical molecular weight ratio of *1.75*, consistent with a dimeric form (Fig. 1F and G). In contrast, treatment with H_2_O_2_ produced an additional peak at fraction 12, corresponding to a molecular weight ratio of *4.27*, indicative of a tetrameric structure (Fig. 1F and G). Notably, the C261S/C573R mutant prevented the H_2_O_2_-induced tetramer formation (Fig. 1F–H), recapitulating the effect of DTT and confirming that disulfides involving C261 and C573 are essential for TRAP1 tetramerization.

### 3.2 TRAP1 tetramers are computationally predicted to form via disulfide bonds between C261 and C573

**Fig. 2:**
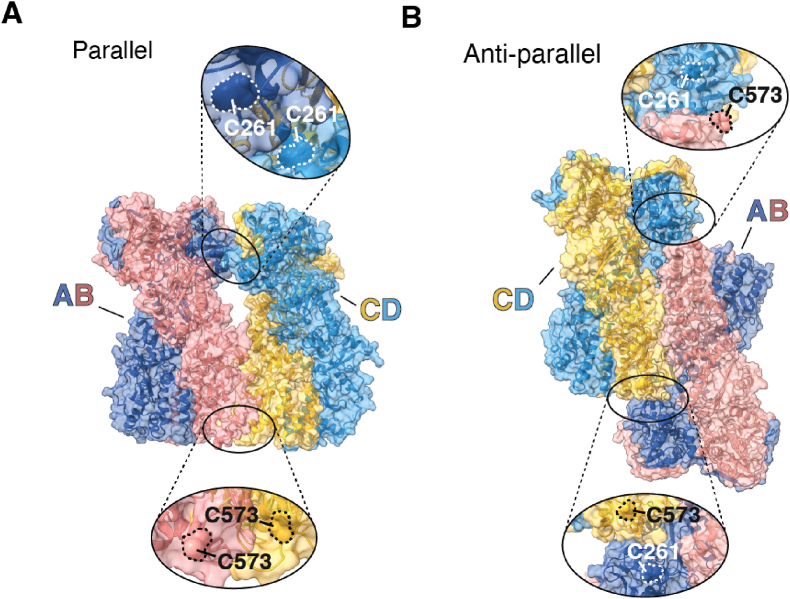
Molecular modeling of TRAP1 tetrameric structures. Structural models of the parallel (**A**) and anti-parallel conformation (**B**) of TRAP1 tetramers. Insets: magnification of C261- and C573-containing regions.

To generate possible structural models recapitulating the assembly of TRAP1 into tetramers compatible with the observed disulfide bridge formation patterns, we used a data-driven docking approach. Recent studies by the Agard group [21] showed that TRAP1 tetramers could form as “dimers-of-dimers”. This was also in line with the evidence that C261 and C573 lie in distant regions, suggesting that any disulfides between these residues should link different TRAP1 dimers. Therefore, we defined the distance between C261 or C573 on one TRAP1 molecule and C261 or C573 on the other as a restraint in protein-protein docking. This bias was introduced to generate final structures in which cysteines on different molecules are within a distance allowing the disulfide formation reaction to occur (also allowing for possible local conformational changes). Using structure 7KLV.pdb as the starting one for each dimer, we applied different combinations of the above reported constraints (see Materials and Methods). The calculations yielded possible structures of tetramers in parallel (Fig. 2A) or antiparallel configurations (Fig. 2B). The first pose of each configuration was optimized through minimization and 10 ns of MD (without any constraints). Interestingly, the parallel configuration is compatible with the establishment of disulfide bonds between C261 of dimer AB and dimer CD and disulfide bonds between C573 of dimer AB and dimer CD (Fig. 2A). Alternatively, disulfide bonds between C261 and C573 of different dimers can be traced back antiparallel configuration (Fig. 2B). Within this frame, the C573R mutation would not only hamper the formation of a covalent disulfide bond but also cause a significant increase in the electrostatic repulsion in the dimer-dimer interface of the tetramer parallel configuration. Although qualitative, these models are compatible with (and reminiscent of) the experimental structures from Agard's group, presenting cysteine residues prompted for disulfide bond formation.

It is reasonable that, at least *in vitro*, both tetrameric forms are generated upon H_2_O_2_ treatment. This could explain why the gel-retarded band is often not well defined.

### 3.3 TRAP1 generates tetramers in cells exposed to oxidative stress

**Fig. 3:**
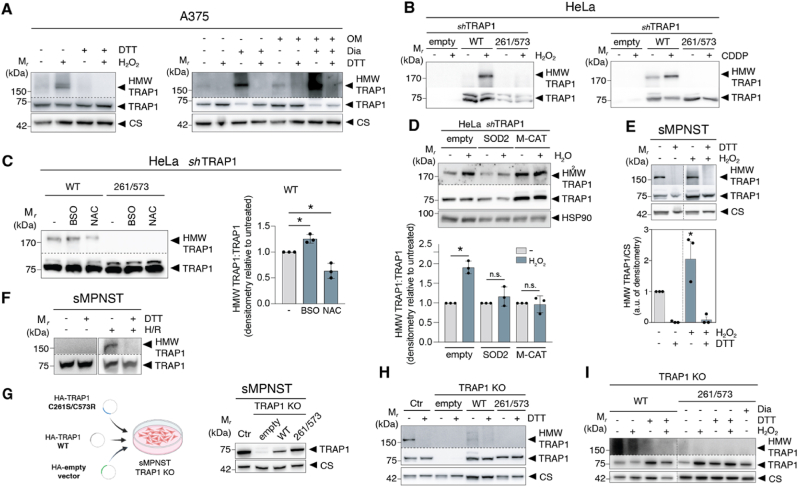
TRAP1 forms tetramers in cells in response to oxidative stress. **A.** Non-reducing Western blot of TRAP1 in mitochondrial extracts of A375 cells treated with 100 μM H_2_O_2_ for 30 min, 1 mM diamide (Dia) for 30 min, or 10 μM oligomycin (OM) for 4 h. Citrate synthase (CS) was used as loading control. DTT was used where indicated to reduce disulfides. HMW TRAP1:high-molecular-weight TRAP1. **B, C.** Non-reducing Western blot of TRAP1 in TRAP1-silenced (*sh*TRAP1) HeLa cells expressing either WT or C261S/C573R HA-linked TRAP1 treated with (**B**) 100 μM H_2_O_2_ or 10 μM cisplatin (CDDP), or (**C**) 100 μM BSO or 10 mM NAC for 24 h. HMW TRAP1:TRAP1 ratio is shown of the right. Data are expressed as densitometry relative to untreated WT cells and represent the mean ± S.D. *t*-test applied for statistical analysis (∗, *p* < 0.05). **D.** (*top*) Non-reducing Western blot of TRAP1 in TRAP1-silenced (*sh*TRAP1) HeLa cells reconstituted with a WT HA-linked TRAP1. Cells were concomitantly transfected with an empty vector, SOD2, or a mitochondria-targeted catalase (M-CAT) and treated with 100 μM H_2_O_2_ for 10 min (*bottom*) HMW TRAP1:TRAP1 ratio. Data are expressed as densitometry relative to untreated WT cells and represent the mean ± S.D. *t*-test applied for statistical analysis (∗, *p* < 0.05; n.s., non significant). **E.** Non-reducing Western blot analysis of TRAP1 in mitochondrial extracts of H_2_O_2_-treated sMPNST cells (*top*). DTT was used where indicated to reduce disulfides. HMW TRAP1 was normalized on citrate synthase (CS) (*bottom*). (n = 3; ∗, *p* < 0.05). Data are expressed as mean ± S.D. *t*-test was applied for statistical analyses. The vertical dotted line indicates that, while the immunoreactive bands were part of the same Western blot, they were not adjacent on the gel. **F.** Non-reducing Western blot analysis of TRAP1 in cell extracts of H_2_O_2_-treated sMPNST cells exposed to hypoxia for 2 h followed by reoxygenation for 10 min (H/R). DTT was used where indicated to reduce disulfides. **G.** Generation of TRAP1-KO sMPNST cells reconstituted with HA-TRAP1 *wild-type*, C261S/C573R mutant, or an empty vector (*top*). Western blot analysis to verify ectopic expression of TRAP1 (*bottom*). Citrate synthase (CS) was used as loading control. **H, I.** Non-reducing Western blot analysis of TRAP1 in mitochondrial extracts of sMPNST cells reconstituted as in D (**E**) and treated with 1 mM diamide (Dia) or 100 μM H_2_O_2_ for 30 min (**F**). Citrate synthase (CS) was used as loading control. DTT was used where indicated to reduce disulfides. The vertical dotted line indicates that, while the immunoreactive bands were part of the same Western blot, they were not adjacent on the gel. Where present, the whole Western blots were reduced in size to show only the high-molecular-weight (HMW) form of TRAP1 and the monomeric TRAP1. A horizontal dotted line stands for this digital cut.

Inspired by these experimental findings and computational predictions, we investigated whether disulfide-mediated TRAP1 oligomerization occurs in cells under oxidative stress. Western blot analysis revealed that H_2_O_2_ treatment induced TRAP1 oligomerization in mitochondrial extract of human A375 melanoma cells, with a high-molecular-weight form of the protein appearing following single or combined treatment with diamide — a compound that promotes disulfide bond formation — and the ATP synthase inhibitor oligomycin (Fig. 3A). In all conditions, this high-molecular-weight form of TRAP1 was not detectable when cells were treated with DTT (Fig. 3A), indicating that it was redox-sensitive and generated through the engagement of disulfide bonds. Collectively, these results suggest that TRAP1 assembles into oligomeric forms in response to various oxidative stress-inducing conditions.

We then examined the role of C261 and C573 in TRAP1 tetramerization by expressing either *wild-type* TRAP1 or the C261S/C573R double mutant in HeLa cells, where endogenous TRAP1 was stably knocked down using specific shRNAs (*sh*TRAP1) (Fig. 3B). Treatment with H_2_O_2_ and the ROS-generating chemotherapeutic agent cisplatin (CDDP) promoted the formation of DTT-sensitive oligomers in HeLa cells expressing *wild-type* TRAP1, but not in cells expressing the double mutant (Fig. 3B). Furthermore, the formation of these DTT-sensitive oligomers could be increased by inhibiting glutathione synthesis with the glutamate-cysteine ligase (GCL) inhibitor buthionine sulfoximine (BSO), or diminished by increasing glutathione levels via *N*-acetyl-l-cysteine (NAC) (Fig. 3C), suggesting that intracellular glutathione levels influence TRAP1 oxidation status. Similarly, ectopic expression of mitochondrial superoxide dismutase (SOD2) or of a mitochondria-targeted form of catalase (M-CAT) did not enhance the formation of TRAP1 DTT-sensitive oligomers following H_2_O_2_ treatment, strengthening the idea that oxidative stress affects TRAP1 functional assembly dynamics (Fig. 3C).

We then extended our study to mouse malignant peripheral nerve sheath tumor (sMPNST) cells, as we previously demonstrated that TRAP1 plays a role in driving the tumorigenic properties of this cancer type [15]. We observed that sMPNST cells displayed significant levels of DTT-sensitive, high-molecular-weight TRAP1 complexes even under basal conditions. This feature was further enhanced upon H_2_O_2_ exposure (Fig. 3E), as previously observed in the other cell types, and in hypoxia/reoxygenation conditions (Fig. 3F). We subsequently generated TRAP1-knockout (KO) sMPNST cells, as previously described [25], and reconstituted them with either the *wild-type* (WT) or the C261S/C573R double mutant TRAP1 (Fig. 3G). Expression of *wild-type* TRAP1 alone was sufficient to induce tetramer formation under basal conditions, suggesting that sMPNST cells experience elevated oxidative stress, which predisposes them to TRAP1 tetramerization even without any exogenous source of ROS. In contrast, cells expressing the C261S/C573R mutant showed no high-molecular-weight TRAP1 structures (Fig. 3H). Oxidative stress induction with H_2_O_2_ increased, as expected, the formation of DTT-sensitive high-molecular complexes in sMPNST cells expressing *wild-type* TRAP1 (Fig. 3I), whereas expression of the C261S/C573R mutant completely abolished this effect under oxidative stress conditions elicited by both H_2_O_2_ and diamide (Fig. 3I). Overall, these findings demonstrate that TRAP1 tetramers are formed in cells through disulfide bond formation between C261 and C573 in response to various oxidative stimuli.

### 3.4 Tetramerization modulates TRAP1 activity and pro-neoplastic functions

**Fig. 4:**
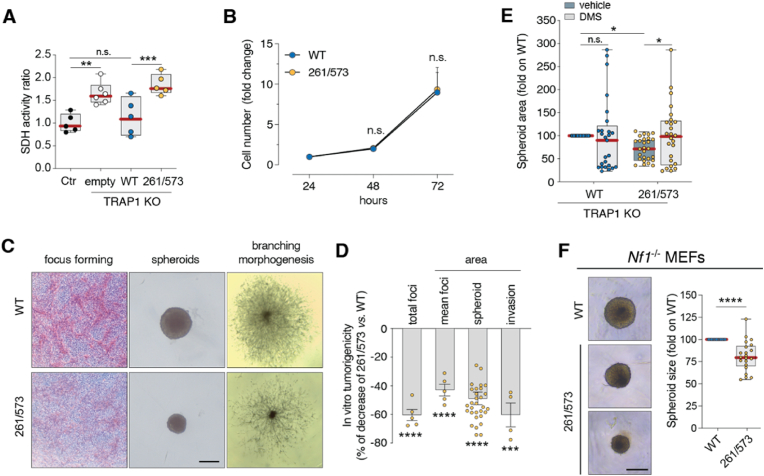
TRAP1 tetramerization contributes to tumor cell aggressiveness. **A.** TRAP1-KO sMPNST cells transfected with HA-TRAP1 *wild-type*, C261S/C573R mutant, or an empty vector were used to assess succinate dehydrogenase (SDH) activity (n = 5; ∗∗, *p* < 0.01; ∗∗∗∗, *p* < 0.0001; *n.s.*, non significant) **B.** Proliferation rate of TRAP1-KO sMPNST cells reconstituted as in A (n = 3; *n.s.*, non significant). Data are expressed as mean ± S.D. Two-way ANOVA was applied for all statistical analyses, unless otherwise indicated. **C.** Representative images of tumor foci, spheroids and invasive masses of TRAP1-KO sMPNST cells reconstituted as in A. Scale bar = 100 μM. **D.** ImageJ evaluation of total foci (n = 5; ∗∗∗∗, *p* < 0.0001); foci area (n = 5; ∗∗∗∗, *p* < 0.0001); spheroid size (n = 30; ∗∗∗∗, *p* < 0.0001), and invasive area (n = 4; ∗∗∗, *p* < 0.001). **E.** TRAP1-KO sMPNST cells transfected with HA-TRAP1 *wild-type*, C261S/C573R mutant, or an empty vector were used to assess spheroid area upon treatment with 2 mM dimethyl succinate (DMS) for 10 days (n = 25; ∗, *p* < 0.05; *n.s.*, non significant — *t*-test analysis). **F.** (*left*) Representative images of tumor spheroids of Neurofibromin 1-deficent (*Nf1*^−/−^) mouse embryonic fibroblasts (MEFs) reconstituted as in C. (*right*) ImageJ evaluation of spheroid size (n = 21; ∗∗∗∗, *p* < 0.0001) was expressed as fold change on WT. Scale bar = 100 μM.

We previously demonstrated that TRAP1 enhances tumor progression by downregulating SDH activity and inducing succinate accumulation [11]. To investigate the role of redox-dependent tetramerization in TRAP1 oncogenic functions, we assessed its modulation of the activity of succinate dehydrogenase (SDH). In sMPNST cells, either TRAP1 ablation or expression of the C261S/C573R TRAP1 mutant, which disrupts tetramer formation, almost doubled SDH activity compared to cells carrying the *wild-type* TRAP1 protein (Fig. 4A), These findings suggest that TRAP1 tetramers play a critical role in modulating interactions with client proteins, exemplified by SDH. Since SDH inhibition results in pro-tumorigenic succinate accumulation, we examined whether the C261S/C573R mutant influences cellular processes linked to neoplastic growth. Although cells expressing either the *wild type* or mutant TRAP1 exhibited comparable proliferation rates (Fig. 4B), those expressing the tetramer-disrupting TRAP1 mutant showed decreased abilities to form foci and their 3D growth in Matrigel, both as spheroids and in branching morphogenesis assays (Fig. 4C and D). Notably, treatment of sMPNST cells expressing the TRAP1 mutant with dimethyl succinate (DMS), a membrane-permeable succinate analogue, rescued their ability to form spheroids of the same size of *wild-type* cells (Fig. 4F), highlighting the importance of TRAP1-depenent modulation of SDH activity in tumorigenic cell growth. Furthermore, the C261S/C573R mutant impaired spheroid formation also in mouse embryonic fibroblasts (MEFs) obtained from mice deficient of the tumor suppressor Neurofibromin (*Nf1*^−/−^) (Fig. 4E), where TRAP1 is hyperactivated by dysregulation of the RAS/MAPK signalling pathway. Taken together, these observations indicate that the expression of the mutant form of TRAP1 in cells hinders their anoikis-resistant invasive growth, hampering *in vitro* tumorigenicity.

### 3.5 TRAP1 tetramerization modulates cellular anti-oxidant response

**Fig. 5:**
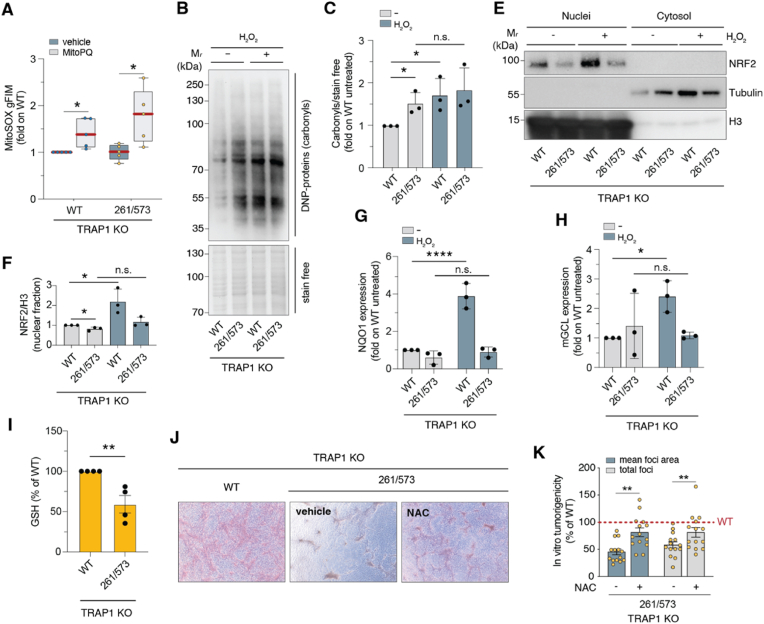
**A. TRAP1 tetramerization contributes to the anti-oxidant response of tumor cells.** TRAP1-KO sMPNST cells transfected with HA-TRAP1 *wild-type* or C261S/C573R mutant were used for MitoSOX evaluation of ROS levels upon treatment with 1 μM mito-paraquat (mito-PQ) for 2 h (n = 5; ∗, *p* < 0.05 — *t*-test analysis). **B.** Western blot of protein carbonyls evaluated in cells as in A. exposed to 400 μM H_2_O_2_ overnight. Lysates were derivatized with dinitrophenylhydrazine (DNP) to detect carbonyl groups on proteins. Stain free was used as loading control. **C.** Densitometry of B. Data are relativized to stain free and expressed as fold on WT untreated cells, and represent the mean ± S.D. *t*-test applied for statistical analysis (n = 3; ∗, *p* < 0.05; n.s., non significant). **D.** Western blot analyses of NRF2 in nuclear and cytosolic fractions of TRAP1-KO sMPNST cells transfected with HA-TRAP1 *wild-type* or C261S/C573R mutant and treated with 400 μM H_2_O_2_ overnight. Tubulin and histone H3 were used as fraction purity controls of the cytosol and nuclei, respectively. **E.** Densitometric analyses of nuclear NRF2 assessed in E. Data expressed as NRF2/H3 and represent the mean ± S.D. *t*-test applied for statistical analysis (n = 3; ∗, *p* < 0.05; n.s., non significant). **F, G.** qRT-PCR analyses of mRNA expression of (**F**) NAD(P)H:quinone oxidoreductase 1 (NQO1) and (**G**) the modulator subunit of glutamate-cysteine ligase (mGCL). Data expressed as fold change on WT untreated, and represent the mean ± S.D. *t*-test applied for statistical analysis (n = 3; ∗, *p* < 0.05; ∗∗∗∗, *p* < 0.0001; n.s., non significant). **H.** Intracellular glutathione (GSH) levels evaluated in TRAP1-KO sMPNST cells transfected with HA-TRAP1 *wild-type* (WT) or C261S/C573R mutant. Data expressed as % of WT and represent the mean ± S.D. *t*-test applied for statistical analysis (n = 3; ∗, *p* < 0.05; ∗∗∗∗, *p* < 0.0001; n.s., non significant). **I.** Representative images of tumor foci of TRAP1-KO sMPNST cells reconstituted as in A, treated with 5 mM N-acetyl-l-cysteine (NAC) every 2 days for 10 days. **J.** ImageJ evaluation of total foci (n = 14; ∗∗, *p* < 0.01), foci area (n = 14; ∗∗, *p* < 0.01). Data are expressed as mean ± S.D. Two-way ANOVA was applied for all statistical analyses, unless otherwise indicated.

Given that TRAP1 provides protection from oxidative stress [28], we further investigated whether tetramers contributed to TRAP1 antioxidant function. As expected, sMPNST cells treated with the redox cycler mito-paraquat (mitoPQ) experienced an increase in mitochondrial superoxide production, which was more pronounced upon reconstitution with the C261S/C573R mutant than with the WT TRAP1 (Fig. 5A). In line with this, sMPNST cells expressing the C261S/C573R mutant accumulated higher levels of protein carbonyls under both basal conditions and following H_2_O_2_ treatment (Fig. 5B and C). sMPNST cells expressing the C261S/C573R mutant were less efficient than WT cells in activating the oxidative-stress sensitive transcription factor NRF2, both under basal conditions and upon H_2_O_2_ treatment, as evidenced by reduced NRF2 nuclear translocation (Fig. 5D and E) and diminished transcription of the modulator subunit of glutamate-cysteine ligase (mGCL) and NAD(P)H:quinone oxidoreductase 1 (NQO1), both selected as NRF2-regulated genes (Fig. 5F and G). Interestingly, sMPNST cells reconstituted with the double mutant show low intracellular levels of GSH (Fig. 5H). Consistent with the notion that efficient antioxidant capacity is essential for sustaining tumor progression in several neoplastic models [12], we observed that expression of the C261S/C573R mutant hindered the focus-forming ability of sMPNST cells, which was almost fully restored by the thiol-reducing agent *N*-acetyl-l-cysteine (NAC) (Fig. 5I and J). All together these results suggest that TRAP1 tetramers work as a “redox antenna” to sense redox environment changes and adapt the anti-oxidant response.

## 4 Discussion

Our findings provide evidence that TRAP1 forms tetramers under oxidative conditions via the formation of disulfide bonds involving C261 and C573 residues, suggesting that TRAP1 senses changes in environmental redox conditions and translates them into structural rearrangements that alter its functional features. Notably, these data expand our previous observations showing that two other cysteines, C501 and C527, are sensitive to oxidants (i.e., nitric oxide) and can form disulfide bridges upon C501 *S*-nitrosylation [17,18], a posttranslational modification leading to changes in TRAP1 stability that influence cell resistance to death stimuli. Combining these two pieces of evidence, we propose that TRAP1 acts as a mitochondrial “redox sensor”, similarly to what has already been described for peroxiredoxins (Prxs) [29]. This could be extremely important for neoplastic cells, where disruption of redox homeostasis is thought to have a major role in disease progression, as both exposure to conditions of dwindling and unstable oxygen supply and dysregulation of several biochemical pathways contribute to ROS surges [8]. According to this, the impact of C261 and C573 mutation on NRF2 activation suggests that TRAP1 could have a broad role in the antioxidant response. We and others have identified in different tumor cell models a number of putative TRAP1 protein clients [13,22], including metabolic enzymes, transporters, OXPHOS components and other chaperones, pointing to the possibility of a multiplicity of TRAP1 interactions under different biological conditions. In this scenario, high-order protein platforms where TRAP1 tetramers could hold together multiple proteins for enabling antioxidant responses is a fascinating option. Furthermore, the possibility that TRAP1 could work as a holdase, in addition to its more conventional chaperone activity [22,30], opens to the possibility that, in the tetrameric assembly, TRAP1 may shield its interacting partners from oxidative damage.

Here we provide direct evidence of a functional connection between subtle changes in TRAP1 and downstream biological events. Our identification of C261 and C573 as being both responsible for TRAP1 tetramerization and required for tumorigenic features of an aggressive neoplastic cell model provides the first example of a structure-function relationship linking TRAP1 chaperone activity to cancer cell biology.

It is well possible that the pro-neoplastic functions of TRAP1 rely upon the differential interaction with several pools of clients, influencing more than one biological routine required for tumor growth. We had previously shown that TRAP1 ablation hampers *in vivo* growth of sMPNST cells [9]. This evidence strongly argues for the importance of TRAP1 chaperone activity in the metabolic rewiring and progression of this aggressive cancer type, for which no treatment currently exists [31]. Here, we further elaborate on this observation, showing that TRAP1 ability to tetramerize is mandatory for two biological processes involved in neoplastic progression, *i.e.* cell capability to overcome anoikis for growing independently of substrate attachment, and invasion of the extracellular matrix, as shown by spheroid formation and branching morphogenesis assays, respectively. Importantly, as a pro-neoplastic role of TRAP1 has been reported in several models [1], it is conceivable that these observations shed light on general mechanisms through which TRAP1 tetramers contributes to tumor growth. This, in turn, raises the possibility of targeting redox-sensitive cysteine residues [32] as a means to selectively interfere with specific TRAP1 functions activated under oxidative stress during neoplastic transformation. While promising, this approach is challenged by the context-dependent reactivity of these cysteines and the risk of off-target effects, highlighting the need for further investigation before therapeutic applications can be realized.

## CRediT authorship contribution statement

**Fiorella Faienza:** Writing – original draft, Validation, Software, Methodology, Investigation, Data curation. **Claudio Laquatra:** Writing – review & editing, Validation, Methodology, Investigation, Formal analysis, Data curation. **Matteo Castelli:** Visualization, Methodology, Formal analysis, Data curation. **Gianmarco Matrullo:** Data curation, Investigation, Validation. **Salvatore Rizza:** Validation, Resources, Data curation. **Federica Guarra:** Software, Methodology, Formal analysis, Data curation. **Azam Roshani Dashtmian:** Methodology, Investigation, Data curation. **Alessia Magro:** Methodology, Investigation, Data curation. **Paola Giglio:** Resources, Methodology, Investigation. **Chiara Pecorari:** Methodology, Investigation, Formal analysis. **Lavinia Ferrone:** Methodology, Investigation, Data curation. **Elisabetta Moroni:** Software, Methodology. **Francesca Pacello:** Methodology, Investigation, Data curation. **Andrea Battistoni:** Writing – review & editing, Writing – original draft, Supervision, Formal analysis, Conceptualization. **Giorgio Colombo:** Writing – review & editing, Writing – original draft, Supervision, Funding acquisition, Formal analysis, Conceptualization. **Andrea Rasola:** Writing – review & editing, Writing – original draft, Supervision, Project administration, Funding acquisition, Formal analysis, Conceptualization. **Giuseppe Filomeni:** Writing – review & editing, Writing – original draft, Visualization, Supervision, Project administration, Investigation, Funding acquisition, Formal analysis, Data curation, Conceptualization.

## Declaration of competing interest

The authors declare that they have no known competing financial interests or personal relationships that could have appeared to influence the work reported in this paper.

## Data Availability

No data was used for the research described in the article.
